# Autogenous Injections in Temporomandibular Disorders: A Systematic Review

**DOI:** 10.3390/jcm14186640

**Published:** 2025-09-20

**Authors:** Sylwia Orzeszek, Andrzej Malysa, Andrej Jenca, Magdalena Gebska, Katarzyna Sluzalec-Wieckiewicz, Marek Zietek, Piotr Seweryn

**Affiliations:** 1 Department of Experimental Dentistry, Wroclaw Medical University, 50-425 Wroclaw, Poland; sylwia.orzeszek@umw.edu.pl (S.O.); andrzej.malysa@umw.edu.pl (A.M.); marek.zietek@umw.edu.pl (M.Z.); 2 Clinic of Stomatology and Maxillofacial Surgery, Faculty of Medicine, University Pavol Josef Šafárik and Akademia Košice, Košice, 041 90 Kosice, Slovakia; andrej.jenca1@upjs.sk; 3 Department of Rehabilitation of Musculoskeletal System, Pomeranian Medical University, 70-204 Szczecin, Poland; magdalena.gebska@pum.edu.pl; 4 Private Dental Practice, 53-407 Wroclaw, Poland; karas60@o2.pl

**Keywords:** temporomandibular disorders, autogenous injections, orofacial pain, platelet-rich plasma, platelet-rich fibrin, injections, intra-muscular, injections, intra-articular, masticatory muscles

## Abstract

**Background/Objectives:** Temporomandibular disorders (TMD) are a heterogeneous group of musculoskeletal conditions affecting the temporomandibular joints and masticatory muscles. In recent years, autogenous injections have been investigated as minimally invasive therapeutic options to alleviate pain and improve function. However, the clinical effectiveness of such therapies across different TMD phenotypes remains uncertain. **Methods:** Electronic searches were performed in MEDLINE, Embase, and Web of Science for articles published between January 2015 and May 2025. Studies involving intra-articular or intra-muscular autogenous injections in TMD patients were included. The risk of bias was assessed using the Cochrane Risk of Bias 2 tool and the Joanna Briggs Institute (JBI) Critical Appraisal Tools. **Results**: Thirteen studies met the inclusion criteria. Six were randomized controlled trials (RCTs) and seven were non-randomized clinical studies. Ten studies evaluated intra-articular conditions such as disc displacement or Temporomandibular Joint (TMJ) osteoarthritis, while three focused on myofascial pain. Platelet-Rich Plasma (PRP) was the most frequently investigated agent. Most studies reported statistically significant reductions in pain and improvements in mandibular mobility following autogenous injections, with PRP generally outperforming comparators such as hyaluronic acid, corticosteroids, or saline. No serious adverse events were reported. **Conclusions**: All PRP and Platelet-Rich Fibrin (PRF) injection protocols reviewed were effective in reducing pain and improving mobility in patients with TMD. However, differences in protocols and follow-up times prevented a meta-analysis from being conducted. More standardized RCTs are needed to determine clear clinical guidelines.

## 1. Introduction

Temporomandibular disorders (TMD) are a complex group of conditions that affect the temporomandibular joint (TMJ) and surrounding muscles, leading to pain and dysfunction [1]. These disorders can significantly reduce quality of life by causing acute or chronic pain, most often in the orofacial region, difficulty with jaw movement, and even psychological distress [2,3,4]. Recent meta-analytical (2024) findings reveal TMD prevalence at 34% within the population [5]. TMD prevalence is projected to rise to 44% by 2050, indicating a growing public health concern [5]. The economic burden of TMD stems from direct healthcare costs, including diagnostics and treatments such as occlusal splints, physiotherapy, and surgery, as well as indirect costs resulting from productivity loss due to pain and functional limitations [5]. These costs vary significantly across regions, influenced by factors such as access to healthcare and insurance coverage, with patients often incurring substantial out-of-pocket expenses where coverage is limited. Addressing the socioeconomic impact of TMD requires strategic public health initiatives and investments in innovative treatments to improve management and reduce long-term costs [4,5]. Among various treatment options, autogenous injection therapies have gained popularity because they are minimally invasive and may provide rapid symptom relief [3,6].

The first approved temporomandibular injections appeared in 1989 [7,8]. Over the past ten years, various injection substances have been used in the treatment of TMD to relieve symptoms and promote healing [3]. These injection therapies vary in effectiveness, often depending on individual patient factors and the specific nature of their TMD [6,9,10]. The choice of substance may depend on the severity of symptoms, the underlying cause of the disorder, and the patient’s overall health profile [6,10]. Biomimetic injectable substances are typically based on hyaluronic acid, which is used to enhance lubrication, restore the normal viscosity of synovial fluid, and provide cushioning for the TMJ. Corticosteroids provide rapid anti-inflammatory effects, especially in acute cases of TMD [11,12]. While they can offer immediate relief, their use is typically limited due to potential side effects with repeated administration [13]. Autogenous injections are becoming a promising option for these substances, utilizing the healing properties of growth factors derived from the patient’s own tissue [11,14]. Autogenous injection generally represents a significant advance in regenerative therapies, leveraging the body’s cells or tissues to promote healing and recovery [12]. This technique reduces the risk of rejection and complications related to foreign materials, making it a promising option for various medical conditions [13].

Platelet-rich plasma (PRP) and platelet-rich fibrin (PRF) are both autogenous injections derived from a patient’s own blood, rich in various growth factors that play crucial roles in healing and tissue regeneration [15]. The specific growth factors found in PRP and PRF include Platelet-Derived Growth Factor (PDGF), which is essential for cell proliferation and plays a significant role in the healing process by attracting stem cells and promoting angiogenesis [16,17]. Transforming Growth Factor Beta (TGF-β) plays a crucial role in regulating cell growth and differentiation, particularly in the formation of connective tissue and the synthesis of collagen, making it vital for tissue repair [16,18]. Insulin-like Growth Factor (IGF), which supports cell growth and development, aids in repairing damaged tissues by promoting the proliferation of various cell types. Epidermal Growth Factor, involved in cell growth and differentiation, is also important [18]. The key difference between the two substances is the concentration of the mentioned factors. For PRP, TGF-β can range from 23.62 to 64.74 pg/mL, whereas PRF contains between 26.21 and 35.80. Within the PRF, growth factors and cells are distributed unevenly. The concentration of platelets and mononuclear cells is higher near the yellow-red interface, whereas polynucleated cells are primarily located below this interface [19,20,21,22,23]. Both PRP and PRF utilize these growth factors to enhance healing, reduce inflammation, and promote tissue regeneration in conditions such as TMD, thereby providing symptomatic relief and improving function [6,10,12].

Autogenous injection, especially PRP and PRF, in the treatment of TMD, represents a promising frontier in regenerative medicine [13,15]. By harnessing the potent growth factors intrinsic to these therapies, clinicians can potentially enhance tissue healing, reduce inflammation, and restore function in affected areas. These biologically active substances not only promote tissue regeneration but also may contribute to improved overall quality of life for patients suffering from chronic pain and dysfunction associated with TMD. The use of autogenous injection treatment is primarily affected by inconsistencies in clinical protocols, differences in methodologies, and the absence of large-scale studies that could help establish standardized practices. Many practitioners may lack adequate training in the techniques required for effective autologous cell extraction and injection. Additionally, there is a lack of consensus on platelet concentration and activation, as well as the specific conditions that benefit from such treatments. This gap can lead to variability in patient outcomes and may hinder wider acceptance and integration of autogenous injection therapies in mainstream medicine. As research continues to elucidate their roles and optimize their use, autogenous treatment is likely to remain an integral component of modern therapeutic strategies aimed at managing TMD effectively [15,16,17,18].

This systematic review focused on studies investigating the use of autologous injections for treating TMD. The primary hypothesis of this review is that autologous injections are effective in alleviating pain and enhancing mandibular mobility in patients with TMD. The following paper will compare the outcomes from various clinical studies to assess this hypothesis.

## 2. Materials and Methods

### 2.1. Protocol and Registration

This systematic review was conducted in accordance with the Preferred Reporting Items for Systematic Reviews and Meta-Analyses 2020 (PRISMA 2020) [24]. The protocol was registered in the PROSPERO database under the registration number CRD420251006942.

### 2.2. Eligibility Criteria

Studies were selected based on the PICOS framework [25]. The following inclusion criteria were applied: (P) adult patients diagnosed with TMD based on Diagnostic Criteria for Temporomandibular Disorders (DC/TMD) [26], Research Diagnostic Criteria for Temporomandibular Disorders (RDC/TMD) [27] or International Classification for Orofacial Pain (ICOP) [28] diagnostic criteria; (I) autogenous intra-articular or intra-muscular injections, including platelet-rich plasma (PRP), platelet-rich fibrin (PRF), injectable PRF (i-PRF), autologous stem cells, or exosomes; (C) comparators included no treatment, placebo, or other forms of treatment such as hyaluronic acid (HA), corticosteroids, physical therapy, dry needling (DN), or non-steroidal anti-inflammatory drugs (NSAIDS); (O) outcomes included pain intensity—e.g., Visual Analogue Scale (VAS) or Numeric Rating Scale (NRS)—mandibular mobility (e.g., maximum mouth opening), quality of life measures (e.g., Satisfaction with Life Scale), and reported adverse effects (e.g., pain, swelling); (S) study designs eligible for inclusion were randomized controlled trials (RCT), non-randomized controlled trials, prospective or retrospective cohort studies, case control studies and cross-sectional studies.

The following exclusion criteria were applied: animal studies; studies involving patients with systemic diseases that could significantly affect healing or inflammatory responses, such as rheumatoid arthritis, psoriatic arthritis, systemic lupus erythematosus, ankylosing spondylitis, Parkinson’s disease, multiple sclerosis, or any form of cancer; case reports, case series, and reviews were excluded. The eligibility criteria are summarized in Table 1.

### 2.3. Search Strategy

A comprehensive search was conducted across electronic databases, including MEDLINE, Embase, and Web of Science. These databases were selected because they provide the most comprehensive coverage of biomedical, clinical, and interdisciplinary knowledge. Together, they ensured a broad and thorough search of the relevant literature. The search process was conducted between 6 April and 31 May 2025. Searches were limited to peer-reviewed journal articles; grey literature was not included. The search encompassed English-language studies published between 1 January 2015 and 1 May 2025. This time frame was selected to capture the most recent and clinically relevant evidence, as autogenous injection techniques for TMD have been primarily investigated within the past decade [3,5]. It ensured the inclusion of contemporary studies while minimizing outdated practices.

The following search string was used in MEDLINE: (“platelet-rich plasma” OR “PRP” OR “platelet-rich fibrin” OR “PRF” OR “autogenous injection” OR “injection” OR “stem cells” OR “exosomes”) AND (“temporomandibular disorders” OR “TMD” OR “temporomandibular joint dysfunction” OR “temporomandibular joint” OR “TMJ pain” OR “TMJ arthralgia” OR “TMJ degenerative disease” OR “masticatory muscles” OR “masticatory muscle pain” OR “masseter muscle” OR “temporal muscle”). Equivalent search strategies were adapted for Embase and Web of Science according to the indexing and syntax of each database.

### 2.4. Selection Process

All identified references were imported into Rayyan QCRI (Qatar Computing Research Institute, Doha, Qatar, and Rayyan Systems, Cambridge, MA, USA) to optimize the screening process [29]. Initially, duplicate records were automatically identified and removed under the supervision of one of the reviewers. The remaining records underwent the following steps: (1) screening of titles and abstracts by two independent and blinded reviewers, supported by Rayyan’s keywords highlighting for potential inclusion or exclusion criteria; (2) full-text assessment of potentially eligible studies; and (3) final inclusion and data extraction. Disagreements were resolved through discussion or by consulting a third reviewer.

### 2.5. Data Collection

The data were extracted by two independent reviewers (P.S. and S.O.) using a standardized form, collecting the following: (1) Author; (2) year of publication; (3) number of participants; (4) study design; (5) assessment method (DC/TMD, ICOP); (6) TMD diagnosis type; (7) Intervention type; (8) Comparator; (9) Outcomes. Outcomes included pain intensity (e.g., VAS, NRS), mandibular mobility (e.g., Maximum Mouth Opening), quality of life measures, and reported adverse effects. As the included studies varied in clinical focus and design, not all outcomes were available across all articles.

### 2.6. Risk of Bias Assessment

For a randomized controlled trial, the Cochrane Risk of Bias 2 (RoB 2) tool was applied [30]. RoB 2 evaluates five domains: (1) randomization process, (2) deviations from intended interventions, (3) missing outcome data, (4) measurement of the outcome, and (5) selection of the reported result. Each domain was judged as “low risk,” “some concerns,” or “high risk,” and an overall risk-of-bias judgment was derived. For non-randomized studies, the appropriate Joanna Briggs Institute (JBI) critical appraisal tool was used, depending on the study design (e.g, cohort, case-control, quasi-experimental), with items assessed as “Yes,” “No,” “Unclear,” or “Not applicable.” [31]. Risk of bias was assessed independently by two reviewers (P.S. and A.M.). Conflicts were resolved through consultation with the third author (S.O.).

### 2.7. Data Synthesis

A narrative synthesis was used to summarize the findings from the included studies. This method was chosen because the studies differed substantially in their design (randomized vs. non-randomized), types of autogenous injections (e.g., PRP, PRF, i-PRF), outcome measures (e.g., VAS, MMO), and follow-up durations. These variations made it inappropriate to conduct a meta-analysis. Therefore, the results were grouped narratively based on the type of intervention and comparator used, and discussed in the context of study design, clinical condition, and outcome domains. This approach allowed for a transparent comparison of effects across studies despite heterogeneity.

## 3. Results

### 3.1. Study Selection

**Figure 1:**
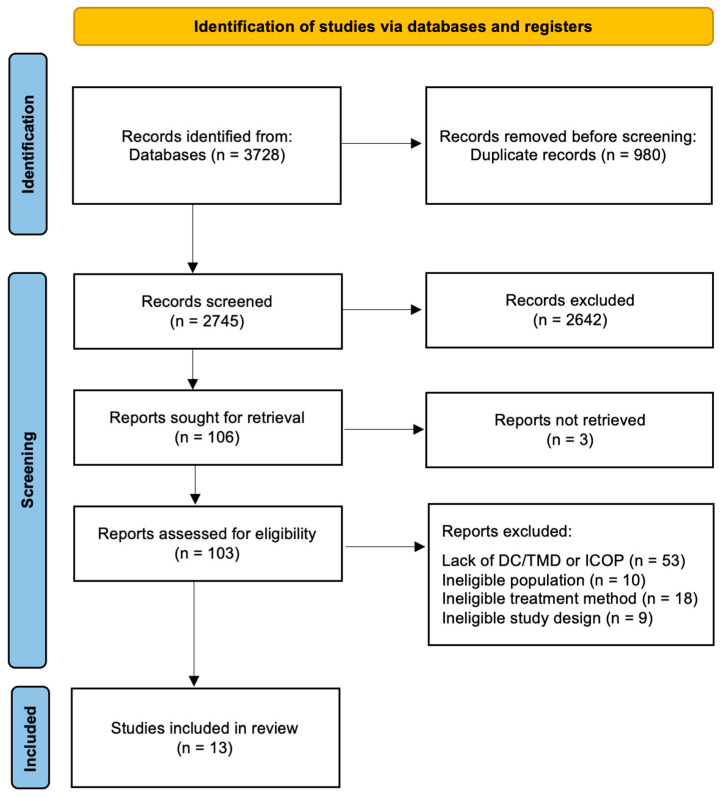
The PRISMA 2020 flow diagram.

A total of 3728 potentially relevant studies were initially retrieved through database searches. After removing 980 duplicates, 2748 titles and abstracts were screened. During this stage, Rayyan’s automatic keyword highlighting tool was used to assist in identifying non-eligible studies. Based on the screening, 106 articles were selected for full-text reading. Following a thorough assessment, 13 studies fulfilled the inclusion criteria and were included in the systematic review [32,33,34,35,36,37,38,39,40,41,42,43,44]. Detailed selection steps are illustrated in Figure 1 [45].

### 3.2. Study Characteristics

A total of 13 studies were included in this systematic review. The studies were published between 2015 and 2024 and involved a combined sample of 926 participants. Sample sizes ranged from 20 to 128 individuals per study.

Among the included studies, six were randomized controlled trials, while the remaining seven were non-randomized clinical studies, including non-randomized controlled trials and retrospective cohort studies. Among the included studies, the most commonly used diagnostic system was the RDC/TMD, applied in nine studies, followed by Di DC/TMD, used in three studies. The ICOP was employed in one study. The included studies addressed both intra-articular and myogenous forms of TMD. Articular conditions encompassed internal derangement, disc displacement with or without reduction, TMJ osteoarthritis, and TMJ pain. Myogenous conditions primarily included myofascial pain and localized masticatory muscle tenderness. Details of the included studies are presented in Table 2 and Table 3.

### 3.3. Risk of Bias

Among the thirteen included studies, the risk of bias was evaluated using tools appropriate to their design. Nine studies were assessed using the Cochrane RoB 2 tool, and four studies were evaluated using the JBI checklist for quasi-experimental studies. Seven studies were rated as having a low risk of bias [33,34,35,36,38,40,41], while six received a moderate risk rating [32,37,39,42,43,44]. The most common issues leading to some concerns of risk of bias were insufficient detail on randomization procedures or unclear bias in the measurement of the outcome. Overall, the methodological quality was considered acceptable, with the majority of studies showing strong internal validity. More detailed analysis of the risk of bias is presented in Table 4.

### 3.4. Evaluation of the Quality of the Evidence

The quality of the evidence presented in the studies, with overall GRADE (Grading of Recommendations Assessment, Development and Evaluation) scores for outcomes, is shown in Table 5.

The studies reviewed in this study were very heterogeneous; therefore, it was not possible to perform a meta-analysis, and instead, a narrative and qualitative summary was prepared. The GRADE approach was used to assess the quality of evidence for outcomes [46]. The quality of evidence was assigned to one of the following categories: very low, low, moderate, or high. Articles classified as high-quality evidence were characterized by a randomized protocol, a detailed description of methodology, and the obtained results. Studies with inaccuracies in methodology, based on a very small sample group, or with a risk of bias were described as moderate. Studies with low quality of evidence lacked complete methodological descriptions, lacked basic information about the size of the study group, and had no randomization protocol or presented a high risk of bias. Six of the thirteen articles qualified for this systematic review were rated as high quality [33,34,38,40,41,44]. The remaining seven papers were rated as moderate due to the small study group, lack of a control group, or suspected risk of bias [32,35,36,37,39,42,43]. None of the studies included were classified as very low quality of evidence.

### 3.5. TMD Type

#### 3.5.1. Myogenous TMD

Three studies focused on patients with muscle-related TMD. Nitecka-Buchta et al. evaluated intra-muscular PRP injections into the masseter muscle and reported a 58.15% reduction in pain intensity by day 5 and a 47.16% reduction by day 14, compared with only 10.24% and 4.62% reductions in the placebo group (*p* < 0.001) [42]. Yilmaz et al. retrospectively compared PRP, botulinum toxin, and local anesthesia for myofascial trigger points in the masseter [43]. All groups showed symptom improvement at one and three months; however, botulinum toxin achieved significantly greater pain reduction and functional improvements lasting up to six months, whereas PRP showed only modest short-term benefits [43]. Agarwal et al. conducted a randomized trial comparing PRP with dry needling and found PRP to be more effective, with greater pain relief and improvement in mandibular function, although precise values were not reported [39]. Collectively, these results suggest that autogenous injections, particularly PRP, provide short-term analgesic effects in myogenous TMD, but botulinum toxin appears more effective in sustaining long-term outcomes.

#### 3.5.2. Articular TMD

Ten studies investigated intra-articular conditions such as disc displacement and temporomandibular joint osteoarthritis. Mathpati et al. demonstrated significant improvements with PRP injections, where mean VAS pain scores decreased from 6.8 ± 1.2 at baseline to 2.1 ± 1.0 after eight weeks (*p* < 0.001), while MMO increased from 38.2 ± 2.5 mm to 43.5 ± 3.1 mm (*p* < 0.001) [34]. Checiński et al. reported that HA resulted in significantly greater gains in mandibular mobility than PRP, with mean improvements in abduction of +4.05 mm (*p* < 0.001) and protrusion of +0.97 mm (*p* = 0.03) [33]. Pihut and Gala observed significant improvements in both PRP and HA groups in disc displacement without reduction, with comparable reductions in VAS pain scores and increases in interincisal distance [41]. Rajput et al. found that both PRP and arthrocentesis significantly improved pain and MMO at twelve months (*p* = 0.0002 and *p* = 0.0022, respectively), though intergroup differences were not significant; PRP showed superiority in reducing joint noise and tenderness [32]. Jamal et al. observed more than 50% pain reduction in seven of ten patients after a single PRP injection, with mean VAS scores decreasing from 7.3 to 3.9 [37]. Kutuk et al. compared PRP, HA, and corticosteroids in TMJ osteoarthritis and reported the largest analgesic effect in the PRP group, with significant reductions in pain between the first and third months (*p* = 0.01) and between the second and third months (*p* = 0.002) [44]. Usman et al. noted greater pain reduction and improvement in MMO after PRP than with corticosteroid injections, although quantitative differences were modest [36]. Vingender et al. compared HA, PRP, and i-PRF for disc displacement, showing significant VAS pain reduction across all groups at six and twelve months (*p* < 0.01), but only the HA group achieved a statistically significant increase in MMO (*p* < 0.01) [35]. Yuce et al. demonstrated that arthrocentesis combined with i-PRF achieved the most pronounced benefits, with significantly greater reductions in pain than the A+HA group at twelve months (*p* < 0.008), and superior improvements in MMO at nine and twelve months [40]. Isik et al. confirmed sustained benefits of i-PRF combined with arthrocentesis in TMJ osteoarthritis, with rapid pain reduction in months 1 and 2 (*p* < 0.001) maintained throughout twelve months, while the control group showed pain recurrence after six months [38].

In summary, intra-articular autogenous injections, particularly PRP and i-PRF, consistently reduced pain and improved mandibular mobility in articular TMD. The greatest and most durable benefits were observed when autogenous preparations were combined with arthrocentesis. Although hyaluronic acid demonstrated superior effects on mandibular mobility in some trials, autogenous injections provided more consistent long-term analgesia and functional stability.

### 3.6. Preparation Type

#### 3.6.1. Injectable Platelet-Rich Fibrin

Vingender et al. examined the effectiveness of platelet-rich fibrin (I-PRF) therapy. The researchers evaluated the efficacy of various substances—hyaluronic acid (HA), PRP, and I-PRF—used in intra-articular injections to treat temporomandibular joint disc displacement. Sixty-eight patients were divided into three groups, and the study measured outcomes such as maximum mouth opening (MMO) and pain levels using the VAS scale. A significant reduction in pain (*p* < 0.01) was observed in all groups after 6 months, with notable differences between 6 and 12 months. HA and PRP demonstrated slightly better results than I-PRF. However, only the HA group showed a statistically significant improvement in MMO (*p* < 0.01). PRP and I-PRF showed improvements in MMO, but these were not statistically significant [35].

Yuce et al. tested three different therapies in their study: arthrocentesis (AO), arthrocentesis plus injection of hyaluronic acid (A+HA), and arthrocentesis plus injection of liquid platelet-rich fibrin (A+I-PRF). The follow-up period was 12 months, during which they assessed results using the VAS scale and MMO. All groups demonstrated a significant decrease in pain levels over time. The A+I-PRF group showed a significantly greater reduction in pain compared to the A+HA group 12 months post-treatment (*p* < 0.008). Both the A+HA and A+I-PRF groups were more effective than the arthrocentesis-only group throughout the observation period. The A+I-PRF group achieved the most tremendous improvement in MMO, with statistically significant differences observed at 9 and 12 months compared to the A+HA group. Therefore, the authors demonstrated the superiority of therapy with an autologous preparation over HA or arthrocentesis alone [40].

Isik et al. also observed similar findings when examining patients with temporomandibular joint osteoarthritis in two groups: the first receiving I-PRF + arthrocentesis, and the control group receiving only arthrocentesis. The follow-up period was 12 months. Clinical assessments included VAS, MMO, and lateral and protrusive jaw movements. Clinical outcomes showed that the I-PRF group experienced rapid and significant pain reduction in months 1 and 2 (*p* < 0.001), which was maintained through 12 months. The control group experienced pain relief up to 6 months, but it recurred by month 12. The difference between the groups was statistically significant throughout the follow-up (*p* < 0.001). In the I-PRF group, MMO and both lateral and protrusive movements significantly improved until month 6 and remained improved through month 12. In the control group, improvements peaked at 6 months but declined by month 12, indicating significant functional loss. The authors concluded that I-PRF preparations combined with arthrocentesis, rather than isolated arthrocentesis alone, especially I-PRF, should be considered a preferred adjunct in the long-term management of TMJ-OA [38].

#### 3.6.2. Platelet-Rich Plasma

Mathpati et al. conducted a prospective, randomized, double-blind, placebo-controlled clinical trial to assess the effectiveness of PRP injections in young adults with mild TMD. The study included 128 participants aged 18–35, randomly assigned to either a PRP treatment group or a placebo group. The PRP group received intra-articular injections of 0.5 mL of PRP per temporomandibular joint, while the placebo group received 0.5 mL of normal saline. The primary outcome measures were TMJ pain (evaluated with the VAS scale), maximum mouth opening (MMO), lateral excursions, and patient-reported outcomes related to daily activities. After eight weeks, the PRP group experienced a statistically significant reduction in pain (VAS score decreased from 6.8 ± 1.2 to 2.1 ± 1.0, *p* < 0.001), along with notable improvements in MMO (from 38.2 ± 2.5 mm to 43.5 ± 3.1 mm, *p* < 0.001) and lateral excursions (from 12.3 ± 1.5 mm to 14.9 ± 2.0 mm, *p* < 0.001). Furthermore, improvements were noted in daily activities, including eating, speaking, and sleeping. The placebo group showed no significant changes. No serious adverse events occurred, confirming the safety and potential of PRP as a minimally invasive therapy for TMJ pain management in young adults [34].

Nitecka-Buchta et al. conducted a randomized, double-blind, placebo-controlled clinical trial to evaluate the analgesic effect of platelet-rich plasma intra-muscular injections in treating myofascial pain within the masseter muscle in patients diagnosed with TMD. A total of 59 patients were randomized into two groups: one receiving PRP injections and the other receiving isotonic saline as a placebo. Each patient underwent three bilateral intra-muscular injections (six sites total, 0.5 mL per site) into the masseter muscles. Pain intensity was assessed using the VAS on days 0, 5, and 14. The PRP group demonstrated a 58,15% reduction in pain on day 5 and a 47,16% reduction on day 14, whereas the placebo group showed reductions of only 10,24% and 4,62%, respectively (*p* < 0.001). Minor, transient side effects such as swelling and muscle soreness were reported. The authors concluded that PRP might be effective in providing short-term analgesic relief in myofascial TMD pain [42].

Checinski et al. conducted a prospective clinical study to compare the effectiveness of PRP and hyaluronic acid intra-articular injections in improving mandibular mobility and reducing joint pain in patients with TMD. A total of 78 patients (128 TMJs) were assigned to two equal groups of 39 patients each, receiving either HA or PRP in five sessions at 7–10 day intervals. Clinical outcomes were assessed using maximum mouth opening (MMO) and the VAS for pain recorded at baseline and follow-up. The HA group demonstrated statistically significant improvements in all directions (*p* < 0.01), while the PRP group did not show significant changes from baseline. Between-group analysis revealed that HA resulted in significantly greater improvements in abduction (MD = −4.05 mm, *p* = 0.00) and protrusion (MD = −0.97 mm, *p* = 0.03). The authors concluded that HA provides superior improvement in mandibular mobility compared to PRP, likely due to its lubricating properties. The study was non-randomized and non-blinded, with a non-concurrent control group [33].

Rajput et al. conducted a prospective clinical study to compare the clinical effectiveness of intra-articular PRP injections with arthrocentesis in patients with groups II and III of RDC/TMD. A total of 24 patients were equally divided into two groups. One group received a single PRP injection into the superior joint space, while the other group received arthrocentesis with Ringer’s lactate. Clinical assessments were performed over a 12-month period, evaluating pain (VAS), painless and maximum mouth opening, lateral jaw movement, and joint tenderness. In both groups, intragroup comparisons revealed statistically significant improvements in pain and mandibular mobility. In the arthrocentesis group, VAS scores improved significantly (*p* = 0.0001), and in the PRP group, the improvement was also significant (*p* = 0.0002). Maximum mouth opening was also significantly enhanced, with *p* = 0.0010 for arthrocentesis and *p* = 0.0022 for PRP. However, intergroup comparisons showed no statistically significant differences in either pain reduction or jaw mobility between the two treatment modalities (*p* > 0.05). Arthrocentesis yielded slightly better results in terms of pain relief and mouth opening, while PRP was more effective in reducing joint noise, deviation, and tenderness. No adverse effects were reported [32].

Kutuk et al. examined the clinical effects of intra-articular PRP, hyaluronic acid, and corticosteroid (CS) injections in the treatment of temporomandibular joint osteoarthritis (TMJ-OA). Sixty patients diagnosed with TMJ-OA were randomly assigned to three treatment groups and received 1 mL injections monthly for three months. Pain (VAS), crepitation, loss of function, and muscle strength were assessed on a monthly basis. A Friedman test showed a statistically significant reduction in pain over time across all treatment groups combined (*p* < 0.0028), confirming the overall effectiveness of intra-articular therapy. Among the three treatments, the PRP group experienced the greatest pain reduction, with significant improvements observed between the first and third months (*p* = 0.01) and between the second and third months (*p* = 0.002). Although HA also showed a significant analgesic effect, CS injections demonstrated limited efficacy. No serious adverse effects were reported during the study [44].

Yilmaz et al.’s study aimed to evaluate and compare the effectiveness of local anesthesia (LA), botulinum toxin (BTX), and PRP injections for treating myofascial trigger points (TrPs) in the masseter muscle. Conducted as a retrospective study, it included 82 patients with myofascial TrPs in the masseter muscle, divided into three groups: 27 patients received LA injections, 26 received BTX injections, and 29 received PRP injections. The findings showed that all treatment methods improved symptoms at both 1 and 3 months. However, BTX injections were superior, showing significant improvements in pain, jaw function, and quality of life up to 6 months. LA injections were effective in the short term, while PRP showed less improvement compared to the other methods. The study concluded that BTX injections offer longer-lasting relief for myofascial TrPs in the masseter muscle [43].

Jamal et al. conducted a prospective observational clinical trial involving 10 participants (9 women and 1 man) aged between 27 and 75 years, who suffered from painful dysfunction of the temporomandibular joint (TMJ). The study aimed to assess the effectiveness of a single PRP injection in reducing joint pain and improving mandibular motion. The study revealed that 7 out of 10 patients experienced a significant decrease in pain intensity (more than a 50% reduction), 2 patients showed partial symptom resolution, and 1 patient showed no improvement and subsequently underwent arthrocentesis. The average pain score decreased from 7.3 preoperatively to 3.9 postoperatively. The study concluded that PRP injections have a statistically significant and strong correlation with pain reduction, supporting their role as an effective treatment for TMJ internal derangement [37].

Pihut with Gala study focuses on the effectiveness of two different intra-articular injection treatments, PRP and HA, for patients suffering from disc displacement without reduction. The study was designed as a double-blind clinical trial involving 100 patients diagnosed with TMD (IIb according to the RDC/TMD). Participants were alternately assigned to either the PRP group or the HA group, with 50 patients in each. The sample included a mix of genders, with the PRP group comprising 38 females and 12 males, while the HA group included 40 females and 10 males. All patients reported symptoms persisting for 10 days to 2 months prior to treatment, and each underwent a comprehensive functional examination of the masticatory system, assessing parameters such as maximal interincisal distance and pain intensity using a VAS. Both groups received intra-articular injections of either 0.4 mL of PRP or 0.4 mL of HA into the temporomandibular joint. This procedure was repeated three times at 10-day intervals. The outcomes indicated that both treatments resulted in significant improvements in pain and function, suggesting that both PRP and HA are effective options for managing TMD related to disc displacement without reduction [41].

Agarwal et al. investigated the comparative effectiveness of PRP injection and dry needling for TrPs in the Masseter Muscle. The randomized controlled trial included 30 participants. Patients in the test group received a PRP injection of 0.5 mL per trigger point (TrP) in the masseter muscle using a 1.5-inch, 27-gauge needle. In contrast, the control group underwent dry needling (DN) at the TrPs in the masseter muscle with the same needle size, but without any solution. Based on the study’s findings, it can be concluded that PRP appears to be more effective than DN in treating myofascial trigger points (MTrPs) in patients with myofascial pain syndrome [39].

## 4. Discussion

Patients with TMD often experience masticatory muscle pain and limited mouth opening, which, in advanced cases, can significantly hinder daily activities [47,48]. TMD may reduce quality of life and place a broader burden on the healthcare system [49]. Current TMD treatments aim to relieve pain and restore normal TMJ function. One rapidly growing treatment option for TMD is intra-articular injection therapy. Its increasing popularity is due to its minimally invasive nature and proven effectiveness in quickly decreasing joint pain and improving mandibular movement [47,48,49,50].

From an economic standpoint, the cost of PRP/PRF treatments can vary significantly based on the provider, geographic location, and specific protocols used [4]. Often, these treatments can be expensive and may not be covered by insurance, which could limit accessibility for some patients. Patients must weigh the potential benefits against the financial burden, especially if multiple treatment sessions are required. Accessibility also plays a crucial role [4,5]. Not all clinics offer PRP/PRF treatments, and patients may need to travel to specialized facilities, which could pose additional costs and challenges. Furthermore, the frequency and duration of treatment can affect patient compliance and overall effectiveness [6,9].

Concomitant treatments, including the use of splints and medications, alongside patient characteristics such as age, gender, and disease duration, can have a considerable impact on treatment outcomes for TMD [51]. These factors can influence how patients respond to various interventions, potentially leading to differing efficacy rates among individuals. Splints and medications play a crucial role in influencing treatment outcomes for TMD [52]. Occlusal splints are designed to alleviate jaw tension and stabilize the bite, which can lead to decreased muscle strain and enhanced comfort for patients [52]. Medications play a pivotal role in managing the symptoms of TMD, providing relief from pain and inflammation associated with this condition. For instance, non-steroidal anti-inflammatory drugs, muscle relaxants, and corticosteroids can provide symptomatic relief by reducing inflammation, making them first-line options for many patients’ muscles [53]. The effectiveness of these treatments can vary based on individual patient characteristics, including the specific TMD phenotype being treated, the duration of symptoms, and concurrent health conditions.

Commonly recognized phenotypes include myogenic, arthrogenic, and mixed types [9,13]. Myogenic TMD is primarily related to muscle dysfunction and pain, often associated with bruxism or jaw clenching. Arthrogenic TMD, on the other hand, involves joint-related issues, such as internal derangements or osteoarthritis of the temporomandibular joint (TMJ). Mixed types exhibit characteristics of both muscle and joint dysfunction [13].

The most common scale used across all reviewed studies was VAS. Main limitations of the VAS include its reliance on the patient’s subjective interpretation of pain, which may vary based on individual differences in pain perception and expression. Additionally, VAS may not adequately capture the multidimensional nature of pain, such as its emotional or psychological components [54].

### 4.1. Platelet-Rich Plasma

Platelet-rich plasma is an autologous blood-derived product obtained through centrifugation of the patient’s peripheral blood. The final product is a plasma concentrate with a high platelet count and an abundance of growth factors such as PDGF, TGF-β, vascular endothelial growth factor (VEGF), epidermal growth factor (EGF), and IGF, which play key roles in tissue regeneration and repair [55,56]. Different PRP preparation methods result in compositional and therapeutic discrepancies. From the clinical perspective, the lack of standardization hampers the comparison of results from clinical trials that may have employed different protocols for PRP production. This fact may explain the heterogeneity of results observed and contribute to the uncertainties related to the clinical effects of PRP [57,58]. Platelet-rich plasma is widely used due to its anti-inflammatory effects and ability to promote tissue regeneration. Leukocyte-rich PRP (LR-PRP) can enhance acute-phase inflammation, aiding tissue repair [59,60]. However, excessive platelet levels may impair tissue repair [61]. These variations in PRP composition significantly impact clinical outcomes.

In the management of TMD, the PRP injection technique involves intra-articular administration of the prepared concentrate directly into the temporomandibular joint space. PRP exhibits anti-inflammatory, analgesic, and regenerative properties, making it a promising alternative or adjunct to conventional conservative treatments for temporomandibular joint osteoarthritis (TMJOA) [62,63]. These mechanisms are not fully understood and may depend on growth factor-mediated pathways [57,58].

The studies reviewed demonstrate several benefits of using PRP in intra-articular injections. There is a significant reduction in pain and improvement in jaw function [33,34,37,44]. Additionally, there is a decrease in myofascial pain and a reduction of myofascial trigger points in the masseter [39,42]. PRP has proven more effective in alleviating joint noise, deviation, and tenderness [32]. In summary, PRP therapy appears to be a promising, effective, and minimally invasive option for managing various TMD-related conditions. However, its effectiveness may vary depending on the specific disorder and treatment approach. Furthermore, PRP injections are described as a very safe procedure, associated with a low risk of complications, making it a desirable treatment option.

### 4.2. Platelet-Rich Fibrin

Injectable Platelet-Rich Fibrin is a second-generation autologous platelet concentrate, developed to overcome some of the limitations of traditional PRP and solid PRF. Solid platelet-rich fibrin (PRF), in its membrane or clot form, presents several limitations that may restrict its clinical applicability in dentistry. Due to its solid consistency, it cannot be injected into anatomically constrained sites, such as narrow bony defects or intra-articular spaces, where a liquid formulation like injectable PRF (i-PRF) may offer superior adaptability. Furthermore, solid PRF must be applied immediately after centrifugation, as the fibrin matrix rapidly dehydrates, leading to loss of biological activity. Finally, the dense fibrin architecture may impede the uniform diffusion of growth factors, potentially resulting in a less homogeneous and temporally restricted regenerative stimulus when compared with liquid platelet concentrates [64]. Unlike solid PRF, which forms a fibrin clot during centrifugation, i-PRF remains in liquid form for several minutes after preparation, allowing for injectable use and more versatile clinical applications [65]. The i-PRF preparation protocol is based on the Low-Speed Centrifugation Concept (LSCC), which helps preserve higher concentrations of platelets and leukocytes in the upper plasma layer, while maintaining bioactive molecules in a more physiologically favorable fibrin matrix [66,67]. Due to the absence of anticoagulants, i-PRF begins to polymerize slowly after injection, forming a fibrin scaffold in situ. This scaffold facilitates sustained release of growth factors, such as PDGF, TGF-β, and vascular endothelial growth factor (VEGF), over several days [68]. These bioactive molecules play essential roles in promoting angiogenesis, reducing inflammation, and stimulating tissue regeneration [69]. In the field of temporomandibular joint disorders, i-PRF has demonstrated its potential to reduce joint pain, improve mandibular mobility, and promote cartilage regeneration through its regenerative and anti-inflammatory properties [70,71].

The studies we analyzed proved the effectiveness of injection therapies using autologous preparations in patients with TMD. The authors of the studies describe the following effects after using PRF: increased mouth opening range and reduced pain [35,38,40]. Additionally, in the long-term management of TMD, PRP and I-PRF support the repair of damaged tissues by releasing growth factors, including PDGF, TGF-β, and VEGF [35,38,40]. The preparations are derived from the patient’s blood, eliminating the risks of allergic reactions, rejection, or disease transmission [38,40]. As a result, these preparations are associated with a very low complication rate, mainly mild, localized symptoms such as pain or swelling, without any serious effects. The procedure is simple and performed on an outpatient basis [35,38].

In addition to techniques using autologous preparations for intra-articular injections in patients with temporomandibular joint dysfunction, several scientific studies have also explored the technique of isolated arthrocentesis and intra-articular injections of preparations such as hyaluronic acid, collagen, or glucocorticosteroids.

### 4.3. Arthrocentesis

Arthrocentesis of the TMJ is a minimally invasive procedure that involves lavage of the joint space using a physiological solution, typically achieved by inserting two needles into the upper joint compartment. This technique is beneficial in the management of TMJ functional disorders, especially in cases of limited mandibular mobility, joint pain, and disc displacement without reduction (most beneficial in early stages of displacement without reduction) [72].

Arthrocentesis can also serve as a method of access for intra-articular injections of anti-inflammatory drugs, hyaluronic acid, PRP, or i-PRF [73]. Its mechanisms of action include the reduction in intra-articular pressure, the removal of inflammatory mediators (e.g., prostaglandins, cytokines), the release of adhesions, and the improvement in disc mobility and joint function [74,75].

Clinical studies show high efficacy of arthrocentesis in reducing joint pain and improving mouth opening in patients with TMJ dysfunction, particularly in those unresponsive to conservative therapy [76]. Kim et al. reported that the average MMO increase after arthrocentesis in patients with TMD was 9.10 mm. The average pain relief of patients after arthrocentesis was 3.03 on the VAS scale [77]. Demir et al. in their study evaluated the effect of arthrocentesis in terms of maximum mouth opening and pain in patients with TMD of intra-articular origin. Arthrocentesis was observed to be effective in terms of pain and function in TMJ patients in this study. It was observed that splint therapy, physical therapy, and medical therapy made no additional contribution to arthrocentesis in terms of MMO or pain [78].

### 4.4. Glucocorticosteroids

Corticosteroids are frequently employed as a minimally invasive option for treating internal disorders of the TMJ. Their strong anti-inflammatory properties have been effective in alleviating joint-related pain. These agents work by suppressing the synthesis and release of pro-inflammatory cytokines, as well as limiting the accumulation of immune cells, such as macrophages and neutrophils, at inflammatory sites. Additionally, they prevent the buildup of inflammatory cells, such as macrophages and neutrophils, by downregulating endothelial adhesion molecules and reducing the synthesis of plasminogen activators. Among the corticosteroids most often used for intra-articular applications are hydrocortisone, methylprednisolone, dexamethasone, betamethasone, prednisolone, and triamcinolone [79]. The choice of corticosteroid will depend on the specific clinical situation and should be based on pharmacokinetics, duration of action, and risk to cartilage [80,81].

In the literature, there are reports on the effectiveness of using corticosteroids in treating pain [80,82,83,84,85].

Isacsson et al. also evaluated the effectiveness of a single intra-articular injection of methylprednisolone for pain relief in patients with TMJ arthritis, in comparison with a placebo. The study involved 26 patients in the methylprednisolone group and 26 in the placebo group. The intervention consisted of a single 40 mg intra-articular injection of methylprednisolone. The observation period lasted four weeks. Additionally, both groups received stabilization splint therapy. No significant differences were found between the groups in terms of resting pain or pain during mouth opening. The authors attributed the observed reduction in resting pain to the use of the occlusal splint [86].

Frid et al. assessed the safety and efficacy of intra-articular corticosteroid injections into the TMJ in adolescents with juvenile idiopathic arthritis (JIA), evaluating both clinical outcomes and Magnetic Resonance Imaging (MRI) findings. All patients received concurrent standard systemic treatment, including methotrexate or biological agents. MRI results showed a statistically significant reduction in the mean additive inflammatory score, from 4.4  ±  1.8 to 3.4  ±  2.0 (*p* = 0.040). The maximum incisal opening (MIO) improved slightly, with a mean increase from 44 mm to 45 mm (*p* = 0.045). However, clinical assessment revealed no significant change in pain levels, indicating a lack of correlation between objective imaging improvement and subjective symptom relief. What may be related to the patient’s psychoemotional state and the way he subjectively assesses his well-being. The authors concluded that intra-articular corticosteroid injections are radiologically effective. Nonetheless, they acknowledged a discrepancy between improvement in MRI and the patients’ subjective perception of symptoms [87]. The researchers did not find any serious adverse effects [87]. Torres et al. conducted a comprehensive analysis of the potential clinical application of steroids in injections for patients with TMD in their systematic review. Across 20 randomized trials (810 participants), betamethasone showed the most consistent pain reduction at one to three months, while arthrocentesis combined with dexamethasone was effective up to six months. No corticosteroid demonstrated clear advantages in improving mandibular function or quality of life compared with arthrocentesis alone. Methylprednisolone, however, was linked to more frequent adverse effects, warranting caution in its use. Clinically, corticosteroid injections may be considered for patients with persistent TMJ pain unresponsive to conservative measures, with betamethasone preferred for short- to medium-term relief. Dexamethasone can be useful in combination with arthrocentesis, whereas routine use for functional outcomes is not supported. The authors of the review, despite conducting a thorough analysis of numerous studies, are unable to provide specific application protocols or indicate clear clinical guidelines for the use of steroids in joint injections for patients with TMD. Treatment decisions should weigh potential benefits against risks, costs, and patient preferences, while further long-term trials are needed to strengthen the evidence base [79].

It is therefore worth noting that glucocorticosteroids can be an alternative to intra-articular injections of preparations derived from the patient’s blood in a specific group of patients, namely those with autoimmune diseases. This group of patients, particularly those with rheumatoid arthritis or psoriatic arthritis, will develop a complex of symptoms associated with temporomandibular joint dysfunction. Consequently, it will also be a group of patients in whom injections of autogenous preparations may be contraindicated in some cases [88,89,90].

### 4.5. Hyaluronic Acid

Another substance successfully used in injections into the TMJ is hyaluronic acid.

Baron et al. conducted a prospective, open-label pilot study to evaluate the effects of a single intra-articular injection of cross-linked hyaluronic acid with mannitol (HANOX-M-XL) in 36 patients with temporomandibular joint osteoarthritis (TMJ-OA). Over a 6-month follow-up, significant improvements were observed: pain during chewing decreased from 6.9 to 2.9, and jaw opening increased from 29 mm to 35 mm. No serious side effects occurred. While specific trends suggested that prior intra-articular injection of HA with or greater early pain relief may predict higher satisfaction, no strong statistical predictors were identified. The study supports HANOX-M-XL as a safe, effective, and convenient single-injection option for managing TMJ-OA symptoms, though larger controlled trials are needed to confirm these findings [91].

Hepguler et al. evaluated intra-articular hyaluronic acid (HA, Orthovisc) in a randomized, double-blind, placebo-controlled trial in 38 patients with disc displacement with reduction. Patients received two HA or saline injections one week apart. At both 1 and 6 months, the HA group showed significant improvements in pain, joint sounds, and jaw function compared to the placebo group. Clinical improvement was observed in 89.5% of HA-treated patients at 1 month and 63% at 6 months. Therefore, the short duration of the effect indicates short-term effectiveness and is a certain limitation for the use of HA preparations. No adverse events were reported. The authors of the study report that HA injections are a safe and effective non-surgical option for managing TMJ disorders with disc displacement, offering benefits lasting up to 6 months [92].

Chęciński et al. in their controlled clinical trial compared the effects of a single intra-articular injection of HA and PRP on mandibular mobility in patients with temporomandibular joint disorders. A total of 78 patients were divided equally into two groups (HA vs. PRP). Mandibular movements—mouth opening (abduction), protrusion, and lateral excursions—were measured after treatment. In this study, HA significantly improved mouth opening (+4.05 mm) and protrusion (+0.97 mm) compared to PRP. No significant differences were observed for lateral movements. HA was more effective than PRP in increasing mandibular mobility, particularly in cases of limited mouth opening. These findings may be attributed to the superior lubricating properties of hyaluronic acid. According to the authors, in cases of TMD characterized primarily by reduced mandibular mobility, intra-articular injections of HA appear to be more appropriate than PRP for symptomatic management [33].

Peng et al. investigated the therapeutic effect of a single intra-articular injection combining PRP and HA in patients with temporomandibular joint osteoarthritis. Twelve participants received one injection of a 1:1 PRP–HA mixture and were followed for six months. The treatment resulted in a marked reduction in pain intensity (VAS score decreased from 6.9 to 2.8) and a significant increase in mandibular mobility, with maximum mouth opening improving from 29.4 mm to 36.5 mm (*p* < 0.01). Additionally, MRI data demonstrated that after 6 months of treatment, PRP/HA regenerated the condylar cartilage and reduced disc displacement. The therapy was well-tolerated with no reported adverse effects. However, due to the small study group, larger studies are needed to confirm safety. These findings suggest that the PRP–HA combination may offer a safe and effective option for symptom relief and functional improvement in TMJ-OA [93].

The systematic review by Agostini et al. provides a systematic organization of information regarding the use of HA in injections for patients with TMD. An umbrella review was conducted to evaluate the role of intra-articular hyaluronic acid (HA) injections in the management of TMD. Eighteen systematic reviews were included, thirteen of which analyzed only randomized controlled trials. Overall, the evidence suggested that HA injections may provide pain reduction and functional improvement, although outcomes were not superior to corticosteroid injections. Platelet-derived products often demonstrated more favorable analgesic effects compared to HA. Importantly, HA injections appeared safe, with no serious adverse events reported other than transient pain at the injection site. However, the methodological quality of the included reviews was generally low, with only a small proportion rated as high quality. Consequently, while HA injections represent a promising option for symptom management in TMD, the certainty of the evidence remains limited, and further high-quality randomized trials with longer follow-up are required to confirm their efficacy [94].

### 4.6. Collagen

Sakr et al. compared the effectiveness of intra-articular injections of collagen versus HA in treating anterior disc displacement with reduction of the temporomandibular joint. Clinical outcomes were assessed using a modified Helkimo Index focusing on pain (P) and joint noise (S), at intervals of 1 week, 1 month, and 3 months after the second injection. The results showed significant improvement in both pain and joint noise within each group over time; however, there was no statistically significant difference between the collagen and HA groups at any follow-up point. This study is notable for being the first to investigate intra-articular collagen injections as a treatment for TMD. The findings support the efficacy of both HA and collagen, and suggest that collagen may represent a promising therapeutic option. However, collagen remains the least studied substance and requires further investigation for broader applications [95]. A systematic review of collagen injections in the case of TMD is limited, although the presented studies and clinical trials show promising results. However, more rigorous studies with longer observation periods are necessary to determine the long-term effectiveness and optimal protocols for collagen injections, as well as specific indications for the use of this type of injection in TMD.

Despite promising preliminary results obtained with PRP and PRF injections and the associated benefits in patient care, further research in larger RCTs is needed, focusing on reducing the risk of bias, to better define the benefits, applications, and protocols associated with the use of PRP or PRF in the treatment of temporomandibular joint disease. It would also be extremely valuable to combine autogenous preparations with hyaluronic acid, collagen, or to use only the arthrocentesis procedure.

### 4.7. Limitations of the Evidence

The chosen primary studies vary in diagnoses, sample sizes, injectable medications, treatment protocols, and follow-up periods. Additionally, the fact that grey literature was not searched may have led to the omission of unpublished data, which could introduce publication bias.

Because of the limitations of this systematic review, the findings should be interpreted carefully. Studies often do not include a control group, and when they do, different approaches are used, with specialists having varying levels of experience performing the treatments. For this reason, a meta-analysis was not suitable.

## 5. Conclusions

In summary, all PRP and PRF injection protocols included in this review showed positive outcomes, relieving pain and improving mobility in patients with TMD, especially in short–medium-term follow-up. A meta-analysis was not feasible due to significant heterogeneity among the clinical studies included in this systematic review. Insufficient and inconsistent data were reported for predefined subgroups (such as stratification by age, sex, disease severity, or comorbidity status). In many instances, subgroup data were missing, inadequately reported, or based on small sample sizes, making any statistical pooling unreliable and susceptible to false-positive or false-negative findings. Therefore, more RCTs with similar methodology are needed to clarify this subject better and establish clear indications for the use of specific intra-articular injection agents in particular clinical situations. Arthrocentesis prior to injection also seems to have a beneficial effect on treatment outcomes. Consequently, it is important to study this topic uniformly and compare the clinical effects in terms of efficacy, safety, and long-term joint preservation against established injection therapies and analgesic effects in myogenous TMD with those of other intra-articular and intra-muscular injections, such as corticosteroids, collagen, or hyaluronic acid.

## Figures and Tables

**Table 1:** Inclusion and exclusion criteria.

	Inclusion Criteria	Exclusion Criteria
Population	Adult patients diagnosed with TMD based on DC/TMD, RDC/TMD, or ICOP	Animals, patients with systemic diseases affecting healing or inflammatory response
Intervention	Autogenous intra-articular or intra-muscular injections	-
Comparison	Any or none	-
Outcome	Primary: pain intensity VAS, NRS, mandibular mobility (e.g., Maximum Mouth Opening (MMO)) Secondary: quality of life (e.g., SLS reported adverse effects (e.g., pain, swelling)	-
Study design	Randomized controlled trials, non-randomized controlled trials, prospective and retrospective cohort studies, case-control studies, cross-sectional studies	Case reports, case series, reviews; studies published before 2015; non-English articles

Glossary: TMD—Temporomandibular Disorders; DC/TMD—Diagnostic Criteria of Temporomandibular Disorders; RDC/TMD—Research Diagnostic Criteria of Temporomandibular Disorders; ICOP—International Classification for Orofacial Pain; VAS—Visual Analogue Scale; NRS—Numeric Rating Scale; SLS—Satisfaction with Life Scale; MMO—Maximum Mouth Opening.

**Table 2:** Detailed information of the included studies.

l.p	Author	Year	Number of Participants	Study Design (RCT, Cross-Sectional, etc.)	Assessment Method (DC/TMD, ICOP)	TMD Diagnosis
1	Mathpati et al. [36]	2024	128 (64 PRP, 64 placebo)	Randomized Controlled Trial (double-blinded)	DC/TMD	TMJ pain, diagnosed via DC/TMD
2	Nitecka-Buchta et al. [42]	2019	58 (29 PRP, 29 Placebo)	Randomized Controlled Trial (double-blinded)	RDC/TMD	Myofascial pain within the masseter muscles
3	Checinski et al. [33]	2024	78 (39 PRP, 39 HA)	Controlled Clinical Trial	ICOP (2020)	TMJ pain
4	Vingender et al. [35]	2023	68 (24 PRP, 20 iPRF, 24 HA)	Non-randomized Controlled Trial	RDC/TMD	Internal derangement of the TMJ
5	Rajput et al. [32]	2022	24 (12 PRP, 12 Arthrocentesis)	Randomized prospective study	RDC/TMD	Groups II and III
6	Kutuk et al. [44]	2019	60 (20 PRP, 20 HA, 20 CS).	Randomized Controlled Trial	RDC/TMD	Axis I Group IIIb
7	Usman et al. [36]	2023	24 (12 PRP, 12 Corticosteroid)	Non-randomized Controlled Trial	RDC/TMD	Anterior disc displacement
8	Yuce et al. [40]	2020	47 (17 i-PRF, 14 HA, 16 Arthrocentesis)	Retrospective Cohort Study	RDC/TMD	TMJ internal derangement and TMJ pain
9	Isık et al. [38]	2022	36 (18 i-PRF + Arthrocentesis; 18 arthrocentesis)	Randomized Controlled Trial	DC/TMD	Temporomandibular joint osteoarthritis
10	Yilmaz et al. [43]	2021	82 (27 LA; 26 BTX; 29 PRP)	Retrospective Cohort Study	RDC/TMD	Myofascial pain within the masseter muscles
11	Jamal et al. [37]	2023	10 (PRP)	Controlled Clinical Trial	RDC/TMD	TMJ pain
12	Pihut, Gala [41]	2020	100 (50 PRP, 50 HA)	Randomized Controlled Trial (double-blinded)	RDC/TMD	Disc Displacement without Reduction
13	Agarwal et al. [39]	2022	30 (15 PRP; 15 Dry Needling)	Randomized Controlled Trial	DC/TMD	Myofascial pain within the masseter muscles

PRP—Platelet-Rich Plasma, DC/TMD—Diagnostic Criteria for Temporomandibular Disorders, HA—Hyaluronic Acid, i-PRF—injectable Platelet-Rich Fibrin, TMJ—Temporomandibular Joint, RDC/TMD—Research Diagnostic for Temporomandibular Disorders, LA—Local Anaesthetic.

**Table 3:** Detailed information about the study’s methodology and risk of bias assessment.

l.p	Author	Year	Intervention	Comparison	Outcome	Risk of Bias	A Tool for Risk of Bias
1	Mathpati et al. [34]	2024	PRP injection	Intra-articular injections of normal saline	Significant reduction in TMJ pain in the PRP group	Low Risk	Risk of Bias tool 2
2	Nitecka-Buchta et al. [42]	2019	PRP injections	Intra-muscular isotonic saline injections	Pain intensity reduction (VAS) at day 5 and day 14.	Some concerns	Risk of Bias tool 2
3	Checinski et al. [33]	2024	PRP injection	Five intra-articular injections of 2% HA	No significant mandibular mobility improvement with PRP; significant with HA	Low risk	Appropriate JBI tool *
4	Vingender et al. [35]	2023	PRP injection; i-PRF	HA	All groups showed significant VAS pain reduction at 6 and 12 months; only the HA group showed significant improvement in MMO.	Low risk	Appropriate JBI tool *
5	Rajput et al. [32]	2022	PRP injection	Arthrocentesis	Both groups showed significant improvement in painless mouth opening and reduction in pain. Arthrocentesis yielded slightly better results in terms of pain relief and mouth opening, while PRP was more effective in reducing joint noise, deviation, and tenderness.	Some concerns	Risk of Bias tool 2
6	Kutuk et al. [44]	2019	PRP injection	HA and corticosteroids	Intra-articular PRP injections decreased TMJ palpation pain more effectively compared with the HA and CS groups.	Some concerns	Risk of Bias tool 2
7	Usman et al. [36]	2023	PRP injection	Corticosteroid (triamcinolone 1.5 mL)	Pain reduction, enhanced mouth opening, and slight increases in joint tenderness.	Low risk	Appropriate JBI tool *
8	Yuce et al. [40]	2020	Arthrocentesis plus I-PRF	Arthrocentesis only, arthrocentesis plus HA	A significant decrease in pain and an increase in MMO were observed in all groups. Arthrocentesis + HA and arthrocentesis + iPRF presented greater differences in pain and MMO at all follow-up time points, in comparison to the arthrocentesis alone.	Low risk	Appropriate JBI tool *
9	Isık et al. [38]	2022	i-PRF after arthrocentesis	Arthrocentesis	The pain levels for the i-PRF group were observed to decrease postoperatively at the 1st, 2nd, 3rd, and 6th months, with these decreases in pain levels preserved through to the postoperative 12th month.	Low risk	Risk of Bias tool 2
10	Yilmaz et al. [43]	2021	PRP injection	Group I (LA injection), group II (BTX injection)	Significant pain reduction in all groups. BTX and LA yielded better outcomes in decreasing VAS scores at 3 months, and BTX provided better outcomes at 6 months of follow-up.	Some concerns	Risk of Bias tool 2
11	Jamal et al. [37]	2023	PRP Injection	No comparator	Increased mobility of the mandible and decreased TMJ pain	Some concerns	Risk of Bias tool 2
12	Pihut, Gala [41]	2020	PRP injection	HA	VAS reduced after treatment in both groups; the same occurred with the mobility of the mandible.	Low risk	Risk of Bias tool 2
13	Agarwal et al. [39]	2022	PRP injection	Dry Needling	PRP is a more effective treatment modality compared to Dry Needling for the management of MTrPs	Some concerns	Risk of Bias tool 2

* JBI Critical Appraisal Checklist for Quasi-Experimental Studies (non-randomized experimental studies), PRP—Platelet-rich Fibrin, TMJ—Temporomandibular Joint, VAS—Visual Analog Scale, HA—Hyaluronic Acid, MMO—Maximum Mouth Opening, BTX—Botulinum Toxin, LA—Local Anaesthetic, MTrPs—Myofascial Trigger Points.

**Table 4:** Risk of Bias for randomized studies.

	Random Sequence Generation	Deviations from Intended Interventions	Missing Outcome Data	Measurement of the Outcome	Selection of the Reported Result	Overall Bias
Mathpati et al. [36]	+	+	+	+	+	+
Nitecka-Buchta et al. [42]	+	+	+	+	?	?
Rajput et al. [32]	?	+	+	?	+	?
Kutuk et al. [44]	?	+	+	?	+	?
Isik et al. [38]	+	+	+	+	+	+
Yilmaz et al. [43]	?	+	+	?	+	?
Jamal et al. [37]	?	+	+	?	+	?
Pihut et al. [41]	+	+	+	+	+	+
Agarwal et al. [39]	+	+	+	?	+	?

+ Low risk of bias; ? Some concerns; - High risk of bias.

**Table 5:** Summary findings for the primary outcome and quality of evidence.

Outcome Significance	Author and Year	Quality of Evidence (GRADE)
Autologous injections are effective in alleviating pain and enhancing mandibular mobility in TMD	Mathpati et al., 2024 [36]	++++ high
	Nitecka-Buchta et al., 2019 [42]	+++- moderate due to imprecision
	Checinski et al., 2024 [33]	++++ high
	Vingender et al., 2023 [35]	+++- moderate due to imprecision
	Rajput et al., 2022 [32]	+++- moderate due to risk of bias
	Kutuk et al., 2019 [44]	++++ high
	Usman et al., 2023 [36]	+++- moderate due to imprecision
	Yuce et al., 2020 [40]	++++ high
	Isık et al., 2022 [38]	++++ high
	Yilmaz et al., 2021 [43]	++-- moderate due to imprecision and indirectness
	Jamal et al., 2023 [37]	+++- moderate due to imprecision
	Pihut, Gala 2020 [41]	++++ high
	Agarwal et al., 2022 [39]	+++- moderate due to indirectness

++++ high certainty; +++- moderate certainty; ++-- low certainty.

## Data Availability

No new data were created or analyzed in this study. Data sharing is not applicable to this article.

## Abbreviations

The following abbreviations are used in this manuscript:

PRP	Platelet-Rich Plasma
PRF	Platelet-Rich Fibrin
i-PRF	Injectable Platelet-Rich Fibrin
TMD	Temporomandibular Disorders
TMJ	Temporomandibular Joint
OA	Osteoarthritis
AO	Arthrocentesis
PRISMA	Preferred Reporting Items for Systematic Reviews and Meta-Analyses
PICOS	Population, Intervention, Comparator, Outcome, Study design
PROSPERO	International Prospective Register of Systematic Reviews
JBI	Joanna Briggs Institute
DC/TMD	Diagnostic Criteria for Temporomandibular Disorders
RDC/TMD	Research Diagnostic Criteria for Temporomandibular Disorders
ICOP	International Classification of Orofacial Pain
VAS	Visual Analogue Scale
NRS	Numerical Rating Scale
MMO	Maximum Mouth Opening
HA	Hyaluronic Acid
CS	Corticosteroids
DN	Dry Needling
NSAIDs	Non-Steroidal Anti-Inflammatory Drugs
RCT	Randomized Controlled Trial
PDGF	Platelet-Derived Growth Factor
TGF	Transforming Growth Factor
IGF	Insulin-like Growth Factor
BTX	Botulinum Toxin
LA	Local Anesthetic
QCRI	Qatar Computing Research Institute
